# Medical and Philosophical News

**Published:** 1781-03

**Authors:** 


### SECTION III.

### Medical and Philosophical News.

THE academy of ſciences at Dijon have propoſed the following ſubject for a prize of three hundred livres value: "To determine "with greater preciſion than hath been hitherto "done, the characteriſtic ſymptoms of intermit-"tent fevers, and to point out in the cleareſt "manner the circumſtances in which febrifuge "remedies may be employed with advantage, "and without danger to the ſick." The diſſertations on this ſubject are to be written in French or Latin, and ſent to M. Maret, ſecretary to the academy at Dijon, on or before the 1ſt of April 1782.

---

Hair, bones, and even complete teeth have ſometimes been found in the female ovarium. Facts of this kind, though perhaps of no great practical utility, cannot fail of proving intereſting to the anatomical reader; we ſhall therefore here mention ſome ſingular appearances that lately prefented themfelves to a celebrated profeffor of anatomy in London, on diffecting a female fubject at his lectures.—Upon opening the cavity of the abdomen, the inteftines, bladder, and uterus were feen forming one difeafed flefhy-like mafs; the Fallopian tubes were indurated and difeafed, and in a word, the whole of thefe parts were fo much confufed as not to be eafily diftinguifhable. But the moft extraordinary circumftance appeared on opening into the rectum; for a little above the fphincter were two tumours, if we may fo call them, placed one immediately above the other; the largeft of the two was about two inches in length, an inch in breadth, and of proportional thicknefs; the other was much fmaller. Both were covered by the inner tunic of the inteftine reflected over them, and were each of them fufpended as it were by a pedicle compofed of feveral fibrillæ. On cutting into their fubftance there were found a quantity of hair, and feveral perfect teeth, incifores, bicufpides, and molares.—Nothing is known of the hiftory of the fubject, but from certain appearances fhe was judged to be about thirteen years of age, and the hymen was obferved to be perfect.

In

In a late *Journal de Medecine*, M. Baillet, furgeon to the Hotel Dieu at St. Vallery fur Somme, gives an account of an extraordinary mifplacement of the ftomach and organs of generation in a fœtus that died a few minutes after its birth. At firft view the moft fingular circumftances attending it feemed to be its having no anus, nor any appearance like the organs of generation, excepting a fmall hole, large enough to admit a probe; every where elfe the fkin had the fame uniform appearance as upon the back and abdomen. Under the fkin where Mr. Baillet obferved this perforation, the fat was an inch and a half in thicknefs. Our author, who had ventured to pronounce the child a female, was not a little furprized, on tracing this paffage with his knife, to find that it terminated in a penis, which, with the reft of the male organs of generation, were concealed under this mafs of fat.

Nothing remarkable appeared in the thorax of this child, but on examining the lower belly M. Baillet found that the œfophagus, inftead of terminating in the ftomach, was connected with the large inteftines; after thefe followed the fmall inteftines, and then the ftomach, which was found in the place where the rectum ought to have been, and adhering by the cardia to the coccyx.

---

At a late monthly meeting of the College of Physicians at Paris, M. le Clerc related a case of phthisis pulmonalis, which had come on in consequence of the sudden repulsion of a cutaneous eruption, and advanced with singular rapidity, so that the patient was in the last stage of consumption, although the cough had begun only about a fortnight before.— M. Bourdois de la Motte read the cases of several unfortunate patients who had fallen victims to an improper use of Keyser's pills, and one of the physicians to the Hotel Dieu took this occasion to remark, that at the time when these pills were the most in vogue, a great number of consumptive patients came into that hospital who owed their complaints to that remedy.

---

M. Gerbier, a French physician, having invented a remedy for cancers, it has been lately administered to seven patients at Paris, under the direction of M. Romilais, one of the physicians to to the Hotel Dieu. The refults of thefe trials were, 1. That only one patient feemed to be cured. 2. That two were fomewhat relieved, though far from being cured. 3. That a fourth patient who was afflicted with fcurvy, as well as cancer, was much worfe after taking it; his fcorbutic fymptoms, though very flight at firft, having made a rapid progrefs after he took the medicine, without the cancers being at all relieved by it. 4. That two other patients died, one foon, and the other five months after taking the remedy, though without its appearing that the medicine was in any degree the caufe of their death. 5. That one of the patients feemed to have fallen a victim to her courage, in perfevering in the ufe of a remedy, which from the firft produced only the moft melancholy effects.

This medicine, we are told, proves violently emetic, an effect that might naturally be expected from a remedy compofed chiefly of verdigreafe.

---

M. Alphonfo le Roy, phyfician at Paris, in a cafe of putrid fever, after applying blifters, and employing antifeptics and cordials to no purpofe, had recourfe to phofphorus; two grains of it were diffolved in a fpoonful of linfeed oil, mixed with with two ounces of water. The patient took a ſpoonful of this mixture every hour. In the account of this caſe, publiſhed in the *Gazette de Santé*, we are told, that during eight days previous to the exhibition of this remedy, the patient (a young man of twenty-four) had voided his urine involuntarily, that his pulſe was ſunk, the pupils of his eyes dilated, and his face and extremities cold and inſenſible. He began to take the phoſphorus at night; the next morning his pulſe was ſtronger, and he was in every reſpect better; the day following he voided his fæces and urine naturally. Six of theſe mixtures were given in the ſpace of ſeven days, and the patient gradually recovered.—This is not the firſt inſtance of the internal uſe of phoſphorus. Kunckel is ſaid to have preſcribed it in the form of pills, and ſome German phyſicians have gone ſo far as to give twelve grains of it for a doſe.*

---

The life of the celebrated Linnæus, written by Dr. Poulteney, F. R. S. Phyſician at Blandford, is in the preſs, and will ſpeedily be publiſhed.

